# An Introduction to the Diseases of Infancy

**Published:** 1891-09

**Authors:** 


### Reviews of Books.

---

*An Introduction to the Diseases of Infancy.* By J. W. BALLANTYNE, M.D., F.R.C.P.E. Pp. viii.—242. Edinburgh: OLIVER & BOYD. 1891.

THE chief part of this book is devoted to the consideration of the anatomy and physiology of the infant, and it is intended as an introduction to a work on the diseases of infancy which the author has in hand. We agree with him that a thorough knowledge of anatomy and physiology is essential as a basis of sound pathological and clinical work, and this is especially the case in the study of infantile diseases, where the differences from the adult state are too often ignored. The author has studied the anatomy of infancy by the method of frozen sections, and useful coloured plates of these sections are given. There is a very thorough account of infantile anatomy, and a great deal of information is here gathered together, of which the author's own researches form no unimportant part. This part of the work is well done, and will be valuable, both as a work of reference on anatomical statistics, and to the student of pædiatrics. Especially useful are the accurate measurements given of all parts of the body. Life is given to the dry bones of anatomical details by the way in which their bearing on clinical work is brought out. Directions for the clinical examination of each region of the body are appended to the anatomical description of the part. We are not so satisfied with these directions; they are not sufficiently complete, and should be either expanded or left out. We think, too, that the author is inclined to exaggerate the difficulties of medical practice amongst children. The section of the book on Physiology is not so good as that on Anatomy; it is rather unequal: the physiology of digestion is however well done. The chapter on artificial feeding does not compare favourably with other standard works on the subject, perhaps because the directions given are not always sufficiently precise.

Where so much is good we do not like to find fault, but the last part of the book, entitled "The Physiology and Hygiene of Infancy," we cannot regard as adding to its value.

It requires to be considerably curtailed, and we should seriously advise the author to leave out all the passages which he considers either fine or humorous writing. Personally, we are not amused by stale jokes from the comic papers, and such sentences as the following simply puzzle us. Are they meant to be humorous, or as a serious contribution to the science of pædiatrics? "To frighten children to sleep by telling them ghost stories or threatening them with a visit from the policemam, is a procedure which may be flattering to the ghosts, but is a cruelty to the child and an insult to the police." Or this, about crying, in a discussion on reflex movements: "There is a quaint conceit, according to which, the infant in his first wailing cry laments his heritage of original sin; but whilst it is true that the baby at an early age alas! begins to show tendencies that do not set for righteousness, it is equally true that the first cry is founded upon a physical and not upon a mental condition."

